# TPSC: a module detection method based on topology potential and spectral clustering in weighted networks and its application in gene co-expression module discovery

**DOI:** 10.1186/s12859-021-03964-5

**Published:** 2021-10-25

**Authors:** Yusong Liu, Xiufen Ye, Christina Y. Yu, Wei Shao, Jie Hou, Weixing Feng, Jie Zhang, Kun Huang

**Affiliations:** 1 Collage of Intelligent Systems Science and Engineering, Harbin Engineering University, Harbin, 150001 Heilongjiang China; 2 Indiana University School of Medicine, Indianapolis, IN 46202 USA; 3 Regenstrief Institute, Indianapolis, IN 46202 USA; 4 Department of Biomedical Informatics, The Ohio State University, Columbus, OH 43210 USA

**Keywords:** Gene co-expression network, Module detection, Topology potential, Spectral clustering, Breast cancer

## Abstract

**Background:**

Gene co-expression networks are widely studied in the biomedical field, with algorithms such as WGCNA and lmQCM having been developed to detect co-expressed modules. However, these algorithms have limitations such as insufficient granularity and unbalanced module size, which prevent full acquisition of knowledge from data mining. In addition, it is difficult to incorporate prior knowledge in current co-expression module detection algorithms.

**Results:**

In this paper, we propose a novel module detection algorithm based on topology potential and spectral clustering algorithm to detect co-expressed modules in gene co-expression networks. By testing on TCGA data, our novel method can provide more complete coverage of genes, more balanced module size and finer granularity than current methods in detecting modules with significant overall survival difference. In addition, the proposed algorithm can identify modules by incorporating prior knowledge.

**Conclusion:**

In summary, we developed a method to obtain as much as possible information from networks with increased input coverage and the ability to detect more size-balanced and granular modules. In addition, our method can integrate data from different sources. Our proposed method performs better than current methods with complete coverage of input genes and finer granularity. Moreover, this method is designed not only for gene co-expression networks but can also be applied to any general fully connected weighted network.

## Background

In the past few decades, high-throughput technologies have rapidly advanced and generated tons of biomedical data. Due to the high dimensionality of the biomedical data with complex relationships, it remains a challenge to analyze them in depth. To tackle this issue, the development of network-based methods has become an effective tool in analyzing complex relationships among great number of entities such as genes and proteins. In the area of biomedical data analysis, networks have been widely developed for different types of data, such as gene co-expression networks [1, 2], co-methylation networks [3], protein–protein interaction networks [4] and gene regulatory networks [5].

Among these biomedical networks, gene co-expression network is one of the most widely studied. These networks have been developed to predict new gene functions [6], discover novel disease biomarkers [7, 8], detect genetic variants [9] and many other applications [10]. Gene co-expression networks are constructed by gene expression profiles and in which each gene is represented by a node and the relationship between a pair of gene is represented by a connected edge. The strength of the relationship between genes is usually quantified by the correlation between the expression of two genes. The Pearson correlation coefficient (PCC) is the most commonly used metric, and some nonlinear metrics such as Spearman rank correlation and mutual information have also been adopted [11]. Gene co-expression networks are divided into weighted and unweighted networks. An unweighted gene co-expression network is a traditional binary network, which only keeps relationships between genes above a specified cutoff, while a weighted network is a fully connected network and keeps all relationships as a continuous measurement. Because setting a cutoff will cause the information loss, weighted gene co-expression networks are commonly used in biomedical analysis [12]. To get biologically relevant information, it is necessary to perform module (or community) detection in gene co-expression networks. WGCNA [13] and lmQCM [14] are the most widely used module detection methods to detect co-expression modules. However, both of them have defects such as insufficient granularity, coverage and unbalanced module size, which prevent us from obtaining complete information of the whole genomic data. Moreover, as demonstrated in [15], introducing prior-knowledge to network-based analysis may hold promise for examining the interactions between genes in complex diseases. Unfortunately, the existing WGCNA and lmQCM can only detect gene co-expression modules without the ability to incorporate such prior knowledge.

Based on the above consideration, we improved upon the topology potential and spectral clustering-based method put forward by Wang et al. [16]. Topology potential, a concept in field theory of complex networks, was first presented by Gan et al. [17] in 2009. It is a metric used to determine essentiality of a node in the network by its relationship with others and has been widely used to identify communities in complex networks. In the bioinformatics field, researchers have tried to find essential proteins in protein–protein interaction (PPI) networks by using topology potential [18]. In the definition of topology potential, there is a mass element to describe the properties of nodes, which can integrate information and knowledge other than topology properties. Spectral clustering is a commonly used unsupervised learning method that has been widely applied to detect communities or modules from networks and graphs [19–21]. However, traditional spectral clustering using the graph Laplacian matrix constructed by the degree adjacency matrix of that graph cannot provide sufficient structural information for community detection [16]. Correcting the Laplacian matrix by topology potential is necessary to improve the accuracy for module detection. However, the method provided by [16] was designed for traditional unweighted binary networks and is not applicable to our fully connected weighted gene co-expression network. In addition, correction of Laplacian matrix in [16] does not consider the mass of nodes, which will cause asymmetry in the Laplacian matrix. Therefore, we improved upon a previous method and compared the results with WGCNA and lmQCM in detecting co-expression modules.

**Fig. 1:**
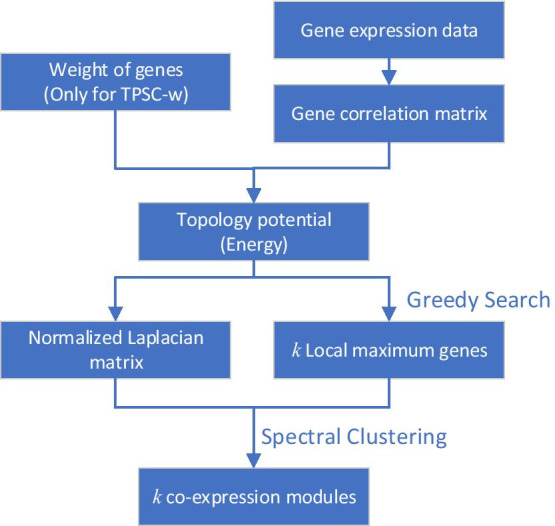
Workflow of TPSC algorithm to detect co-expression modules. TPSC-1 and TPSC-w have similar workflows. The only difference is whether have weight of nodes (i.e. whether introduce prior knowledge)

In this paper, we present the Topology Potential-based Spectral Clustering (TPSC) Algorithm, an improved module detection algorithm based on topology potential and spectral clustering and use it to detect co-expression modules which show significant difference in overall survival time in breast cancer. The workflow of our proposed TPSC algorithm is displayed in Fig. 1. Depending on whether there are weights on nodes, there are two versions of TPSC, with TPSC-1 focusing on networks with only weights on the edge while TPSC-w being able to accommodate weights on both nodes and edges. We compared the results obtained by TPSC-1 with the widely used WGCNA and the lmQCM we previously developed. The experimental results show that our proposed method could detect modules with complete coverage, balanced module sizes and fine granularity. In addition, by introducing weighted nodes, our proposed TPSC-w method could select modules with the guidance of prior knowledge. Our method can mine not only gene co-expression networks, but also any general weighted fully connected network.

## Results

### Choices of ${d}_{min}$

**Fig. 2:**
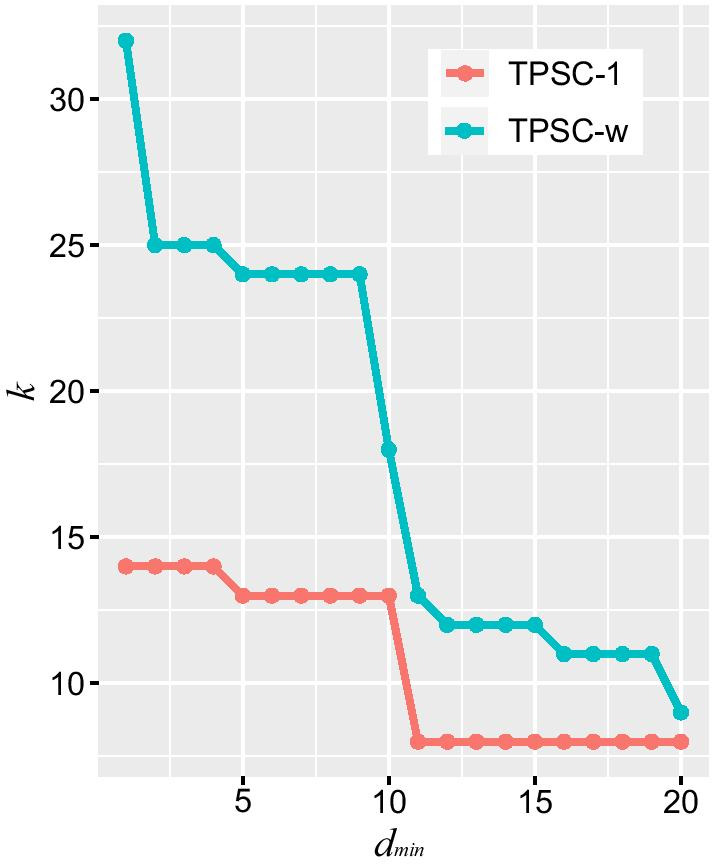
Comparison of the number of modules ($k$) detected by TPSC-1 and TPSC-w with respect to different choice of $d_{min}$. The number of modules $k$ decreased as the parameter $d_{min}$ increased in both TPSC algorithms

In our proposed TPSC algorithm, there are three parameters, namely the minimum degree of local maximum node ${d}_{min}$, the weight cutoff threshold $r$ and the topology potential impact factor $\sigma$. Parameters $r$ and $\sigma$ are determined by properties of the edge weights, which becomes constant once the edge weights are determined. The technical details of choosing these parameters will be discussed in the Discussion section. This also implies only the parameter ${d}_{min}$ will affect the results of our algorithm. The parameter ${d}_{min}$ will directly affect the identified number of modules, and its influence is shown in Fig. 2. It is clear that the number of modules $k$ decreased as the parameter ${d}_{min}$ increased, regardless of with or without weights on the nodes. Compared with the condition of TPSC-1, TPSC-w detected less modules. This make sense since prior knowledge can contribute to the screening for modules. However, in both conditions, the number of modules became stable when ${d}_{min}$ was set between 5 and 9 and we fixed it as 5 in the analysis in the rest of the paper.

### Detected modules

**Fig. 3:**
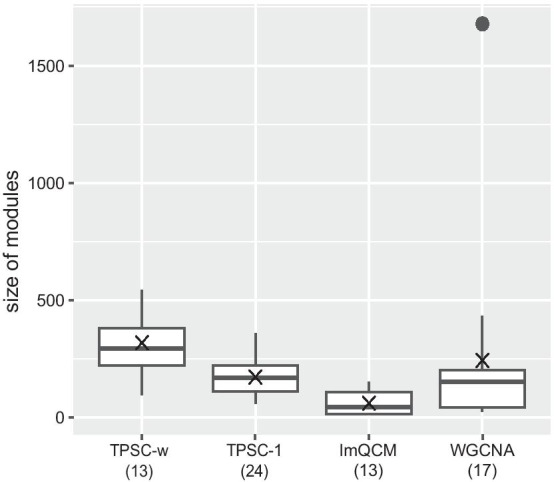
Number and size of modules generated by our proposed TPSC, lmQCM and WGCNA. Numbers marked in parantheses near the name of method describe the number of modules generated by the method, the dot in the figure marks an outlier and the cross describes the average module size of each method

The number and size of modules are important to measure a module detection method. For gene co-expression networks, we do not want to get too many modules as the biological meanings for many of the smaller modules are not clear and it also causes more burden in downstream analysis. We also do not prefer a module with a large number of genes because it is challenging to find out which pathway is the main affect factor. A good module detection method should be balanced between the number and size of modules. In this paper, we compared our proposed method with well-known methods WGCNA and lmQCM (Fig. 3). Parameters for these two algorithms are set based on recommendation in corresponding papers. We show that our proposed method can provide an acceptable number and size of modules regardless of nodes weights.

For the The Cancer Genome Atlas breast cancer (TCGA-BRCA) dataset, we prefiltered the data and 4,125 genes were kept for the tests. The four methods identified 13–24 gene modules with TPSC-1 generated the greatest number of modules (24), meanwhile, lmQCM and TPSC-w generated the least number of modules (13). Introducing weighted nodes decreased the number of modules in our method. The size of modules varied across the four methods. The modules generated by lmQCM are generally smaller than the rest of the other methods, but these modules only covered about a quarter of genes we provided. Modules generated by WGCNA covered all genes, but the method generated a large module with more than 1500 genes, i.e. more than one third of all genes in the network. Compared to lmQCM and WGCNA, TPSC generated modules that not only covered all genes, but also contained a more uniform module size.

**Fig. 4:**
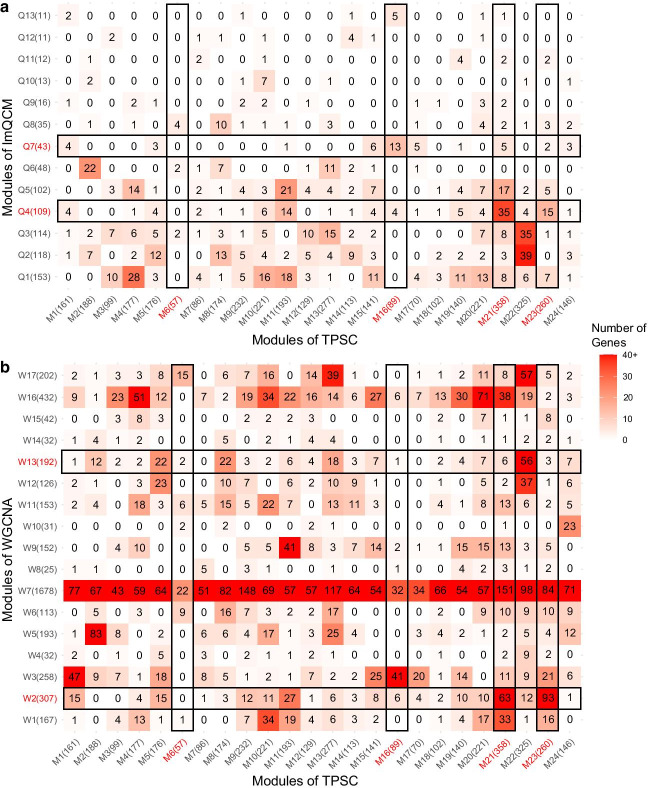
Overlap of modules detected by TPSC-1 with **a** lmQCM and **b** WGCNA. Module size is displayed in the brackets after module name. Numbers of genes that overlapped in both modules is displayed in the heatmap. Modules which showed a significant survival difference are highlighted by red text and surrounded by black boxes

Besides number and size of modules, we also investigated the overlap of modules detected by different algorithms. We compared the overlap of modules detected by TPSC-1 with those detected by lmQCM and WGCNA and the results are displayed in Fig. 4. As we can see in Fig. 4a, only a few modules showed strong overlap between TPSC and lmQCM. This is mainly due to the poor coverage over input genes by lmQCM. However, there were also some modules with strong overlaps, such as Q4 with M21(Fisher’s exact test $p=1.603\times {10}^{-12}$) and Q7 with M16 ($p=1.937\times {10}^{-12}$), which indicates that these two algorithms can also find some common modules with the similar biological implications. In Fig. 4b, we found that the giant module, W7, detected by WGCNA had strong overlap with each module detected by TPSC. Furthermore, there are some modules (such as W2, W3, W16 and W17) detected by WGCNA that showed significant overlap with many modules detected by TPSC, indicating WGCNA lacks granularity in comparison with our method.

**Fig. 5:**
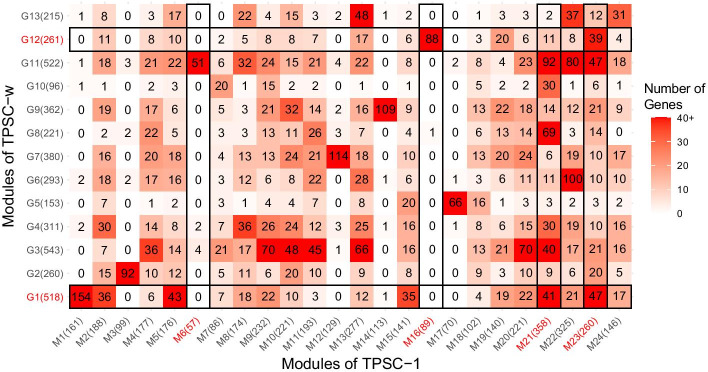
Overlap of modules detected by TPSC-1 with TPSC-w. Module size is displayed in the brackets after module name. Numbers of genes that overlapped in both modules is displayed in the heatmap. Modules which showed a significant survival difference are highlighted by red text and surrounded by black boxes

To investigate how the prior knowledge contributes to modularizing the co-expression network, we also examined the overlaps between modules detected by TPSC-1 with TPSC-w. The results are shown in Fig. 5. While introducing prior knowledge of gene disease associations, some of the modules were kept (such as M3, M14 and M17) but others were divided or merged. Some of the modules detected by TPSC-w integrated multiple modules detected by TPSC-1 (e.g., G1, G3, G11 and G12). These results revealed that prior knowledge of gene disease associations can influence the module detection.

### Survival analysis

**Fig. 6:**
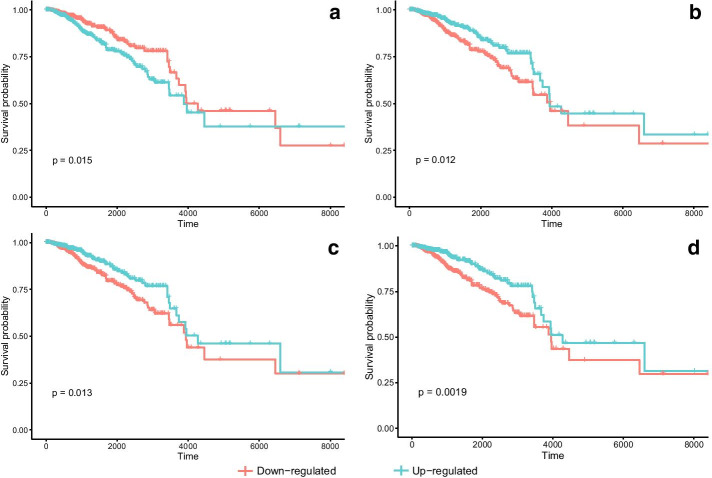
Kaplan–Meier curve of module **a** M21, **b** M23, **c** W2 and **d** Q4

To identify pathways in breast cancer associated with patient survival, we performed survival analysis on the modules detected by the previous four methods. For each module, we divided all 999 samples into two groups by eigengene value. Modules with a positive eigengene value implied potential up-regulation while a negative value means down-regulation. We found four modules showed a significant survival difference in the modules detected by our proposed method without weight of nodes and two modules in each of the other three methods based on Log-rank test *p*-value. It is worthy pointing out that after performing multiple correction by Benjamini and Hochberg false discovery rate (B&H FDR), most of the modules was not significant except for modules G12 and Q4. Kaplan–Meier curve of the cell-cycle related gene module led to significant survival difference detected by TPSC-1, lmQCM and WGCNA (M21, M23, Q4 and W2) is displayed in Fig. 6. Other curves of survival difference significant modules are provided in Additional File 1. Statistic information of these modules is listed in Table 1.

**Table 1:** Statistically significant survival difference of modules generated by four methods

Module	Size	Group with increased Survival	*p* Value (Log-rank test)	*q* Value (B&H FDR)
TPSC-w (2/13)				
G1	518	Downregulated	0.0430	0.2795
G12	261	Upregulated	0.0006	0.0077
TPSC-1 (4/24)				
M6	57	Upregulated	0.0460	0.2760
M16	89	Downregulated	0.0250	0.2000
M21	358	Downregulated	0.0150	0.1800
M23	260	Upregulated	0.0120	0.1800
lmQCM (2/13)				
Q4	109	Upregulated	0.0019	0.0247
Q7	43	Downregulated	0.0280	0.1820
WGCNA (2/17)				
W2	307	Upregulated	0.0130	0.2210
W13	192	Downregulated	0.0490	0.4165

Our proposed TPSC-1 method generated the greatest modules that showed survival difference, but the ratio of number of survival difference significant modules and all modules is similar for all 4 methods. Furthermore, while adding weight of nodes to our method (TPSC-w), the number of modules with significant survival difference is decreased. This may be caused by the weight selection of different nodes.

### Functional enrichment analysis

We performed functional enrichment analysis on the modules with significant survival difference to find out pathways and other biological patterns related to survival. Gene ontology, pathway and cytoband analysis is performed by ToppGene (https://toppgene.cchmc.org/). Table 2 summarized mainly results of biological process, pathways and cytobands enriched in modules detected by TPSC, WGCNA and lmQCM. Detailed enrichment results are displayed in Additional File 2.

**Table 2:** Summary of enrichment analysis on survival significant modules detected by different algorithms

	Biological processes	Pathways	Cytobands
TPSC-w	Cell cycle and mitotic process (G1, G12)	HTLV-I infection and Prostate cancer (G1)	3p21.3 and 8p12-p11 (G1)
		P53 signaling, PI3K-AKt signaling and FOXO signaling pathway (G12)	7q21.3, 1q32 and 17q22-q23 (G12)
			17q11.2(G1, G12)
TPSC-1	Extracellular matrix and structure organization, blood vessel development, cell adhesion and motility (M6)	Extracellular matrix organization, collagen formation and ensemble of genes encoding ECM-associated proteins (M6)	11q22.3 and 13q34 (M6)
	Response to growth hormone (M16)	Pathways related to RHO GTPases (M21, M23)	17q11.2 and 6q22-q23 (M16)
	Cell cycle and mitotic process (M21, M23)	Proteoglycans in cancer and circadian clock (M21)	1p32 (M23)
		Antifolate resistance (M23)	
WGCNA	Cell cycle and mitotic process (W2)	P53 signaling, oocyte meiosis and PI3K-AKt infection (W2)	20q11.2 and 12q13.12 (W2)
	Activation of T cell, lymphocyte and leukocyte (W13)	Cytokine, NF-Kappa B, chemokine and TNF signaling(W13)	1q31-q32, 1p36 and 1q23 (W13)
lmQCM	Cell cycle and mitotic process(Q4)	RHO GTPases pathways, P53 pathways, oocyte meiosis and HTLV-I infection (Q4)	12q13.12, 2p24.2, 15q15.1 and 4q27(Q4)
	Epithelial cell development and mammary gland development (Q7)	ERBB2- and ERBB4-related signaling (Q7)	4q31.21 and 1p33(Q7)

We found two modules that have significant survival difference by using proposed TPSC-w. The first module (G1) was mainly enriched in the biological process of cell cycle and mitosis. This module was also enriched in the pathway of HTLV-I infection and prostate cancer. Furthermore, this module enriched in cytobands 3p21.3, 8p12-p11 and 17q11.2, which are all important chromosomal locations involved for breast cancer. The second module (G12) was also focused on cell cycle and mitotic process. In pathway analysis, P53 signaling, PI3K-AKt signaling and FOXO signaling pathway were enriched. In addition, 17q11.2, 7q21.3, 1q32 and 17q22-q23 were enriched in cytoband analysis.

For TPSC-1, we found four modules with significant survival difference. The first survival significant module (M6) was enriched on the process of extracellular matrix and structure organization, blood vessel development and cell adhesion and motility. This module was enriched in pathways of extracellular matrix organization, collagen formation and ensemble of genes encoding ECM-associated proteins. The second module (M16) was mainly enriched in cytobands on 17q11.2 and 6q22-q23. This module was also enriched in the biology process of response to growth hormone. The other two modules (M21 and M23) were both enriched in cell cycle and mitosis in biological process. They all enriched to pathways related to RHO GTPases. M21 also enriched in the pathway related to proteoglycans in cancer and circadian clock. M23 also enriched in the pathways of antifolate resistance.

As a comparative baseline, we also performed enrichment analysis on modules with significant survival difference generated by WGCNA and lmQCM. The first module generated by lmQCM (Q4) was enriched in cell cycle and mitotic processes. RHO GTPases pathways, *P53* pathways, oocyte meiosis and HTLV-I infection pathways were also enriched. In cytoband analysis, genes in this module was enriched at 12q13.12, 2p24.2, 15q15.1 and 4q27.The second module generated by lmQCM (Q7) focused on epithelial cell development and mammary gland development process. In pathway analysis, this module mainly enriched on ERBB2- and ERBB4-related signaling pathways. 4q31.21 and 1p33 were enriched in cytoband analysis. For modules generated by WGCNA, W2 was also enriched in cell cycle and mitotic processes. W13 was enriched in the process related to immune response: activation of T cell, lymphocyte and leukocyte were the most significant enriched biological processes. Cytokine, NF-Kappa B, chemokine and TNF signaling pathway were especially enriched pathways in pathway enrichment analysis. 1q31-q32, 1p36 and 1q23 was enriched in cytoband analysis.

## Discussion

### Choice of parameters

There are three parameters, i.e., ${d}_{min}, r$ and $\sigma$, in our proposed TPSC algorithm. Here ${d}_{min}$ is a parameter used for determining the number of modules. This parameter limits the number of local maximum nodes under the assumption that central node of a module should have close relationships with other genes in that module. Obviously, increasing ${d}_{min}$ forces local maximum nodes have a closer relationship with other genes and then decreases the number of modules. Since gene co-expression network has local modularity structures, number of local maximum nodes detected should reach a plateau while ${d}_{min}$ increases, indicating a reasonable number of modules. Based on our search method, we will select the local maxima in descending order based on the topology potential of each node. Once the number of local maximum nodes is determined, the selected nodes are also determined. Since these local maximum nodes will become the initial nodes for module detection, the detected modules should be similar. Figure 2 illustrated this situation and the number of modules became stable when ${d}_{min}$ was set between 5 and 9. We therefore empirically set ${d}_{min}$ to be 5.

Both $r$ and $\sigma$ are parameters related to edge weight, so they can be determined by the property of edge weights. Since we used Pearson correlation coefficient (PCC) as weights, we will discuss the condition of using it. In the area of statistical analysis, for two variants, we think they have linear relationship if the absolute value of PCC is greater than a specific value. In the area of biological analysis, the empirical value is 0.3, so we set $r$ and inverse of influence range of topology potential to 0.3. Since the influence range of topology potential can be calculated by $3\sigma /\sqrt{2}$, we can get the value of parameter $\sigma$ as 1.57. No matter the selection of weight and cutoff threshold, we must keep $r$ and inverse of influence range of topology potential as a same value to maintain consistent of module detection and number of clusters determination.

### Coverage and module size

Coverage of input gene list and module size generated are important factors in a module detect algorithm. If a method cannot cover enough genes, it may lose information when generating modules. Additionally, modules whose size are too large or too small may not be informative for the downstream analysis. For instance, large modules will include too many genes which will dilute the information and obscure important findings, while small modules may not contain enough information to reveal its underlying biological meaning. Thus, moderate module size and sufficient coverage are two essential factors for a module detection algorithm.

In our research, our proposed TPSC algorithm found that the module (i.e., M6) related to extracellular matrix and structure organization does not identified by both lmQCM and WGCNA algorithm. We compared the overlap of this module and modules that generated by lmQCM and WGCNA. Only eight genes were found in the modules detected by lmQCM, due to its limitation. For WGCNA, we found there are 22 (38.6%) genes included in the W7, which has 1678 genes. Though overlap between M6 and W7 is not significant ($p=0.6740$), this giant module included lots of pathways and obscured specific pathways of interest. In addition, the module also had 15 genes overlapped with W17 ($p=4.747\times {10}^{-8}$), but W17 didn’t have significant difference on survival because it also showed great overlap with M13 ($p=6.322\times {10}^{-10}$) and M22 ($p=5.079\times {10}^{-19}$) in our proposed TPSC-1 algorithm. This is the problem of granularity which we will discuss in the following section.

In Fig. 3, modules generated by our proposed method had uniform module sizes. For scale-free networks, if we just use node degree as centralization metric, the sizes of detected modules should follow a power law distribution. However, when we include information other than the topology property of the network, such as the GDA score adopted in this paper, the distribution would change. In addition, as discussed by previous research [22], biological network including gene co-expression network is not a completely scale-free network. it obeys to power law distribution in global but subject to modularity locally. This condition also affects the size of detected modules. These conditions turn the module size distribution into uniform. Actually, balanced module size is advantageous for discovering functions and pathways enriched to the modules. Modules with only several genes are hard to analysis due to the lack of information, and huge modules will hide the valuable results under a great number of useless ones. These properties of modules make our proposed method a strong alternative to WGCNA and lmQCM.

### Biological interpretation of the modules

Module detection algorithms can generate gene modules as output, but they do not have the capacity to interpret the modules. Nevertheless, interpreting the modules for their biological significance is crucial in bioinformatics. Enrichment analysis is a widely adopted approach to reveal the biological relevance of the detected modules by comparing the overlap between modules and known gene lists (such as pathways, Gene Ontology terms, literatures, and chromosomal locations such as cytobands). These known lists can be acquired from some various databases (such as Gene Ontology [23], KEGG [24], DAVID [25] and Reactome [26]). In addition, we can also use survival analysis to identify the modules that are strongly associated with patient prognosis and further establish the link between biological relevance (e.g., enriched pathways or biological process terms) with patient outcome.

### Granularity

For biological processes that have significant survival difference, there exists a group of pathways that contribute to it. However, not all these pathways have similar direction of influence. Some pathways are up-regulated while others are down-regulated. If a module detection algorithm can distinguish different directions of pathways that contribute to the same biological process instead just giving out one big module that contain all the pathways, we consider the algorithm can be more granular with more concrete biological insight. Our experimental results showed that TPSC is more granular than WGCNA and lmQCM. All the four methods in our study identified modules related to cell cycle and mitosis (G1, G2, M21, M23, Q4 and W2). Our methods (both TPSC-1 and TPSC-w) found two modules related to cell cycle and mitosis, of which one is up-regulated and the other one was down-regulated. In contrast, under the same condition, both WGCNA and lmQCM only identified one cell cycle related module. From the perspective of overlap between modules generated by different algorithms, we found that both M21 and M23 have substantial overlaps with Q4 ($p=1.603\times {10}^{-12}$ and $3.105\times {10}^{-3}$) and W2 ($p=1.399\times {10}^{-11}$ and $1.260\times {10}^{-43}$). This result suggests that our proposed method can yield more granular modules than WGCNA and lmQCM. On the other hand, module W17 detected by WGCNA has significant overlap with M6 ($p = 4.747 \times 10^{ - 8}$), suggesting extracellular matrix related pathways are included in W17. But unlike M6, genes in W17 were not able to stratify the patients into groups with significant differences in survival time. This is due to the fact that W17 also has great overlap with M13 ($p = 6.322 \times 10^{ - 10}$) and M22 ($p = 5.079 \times 10^{ - 19}$). These extra genes covered pathways not related to patient survival time and thus obscured the signal. These results demonstrate that our proposed algorithm is more granular than WGCNA and lmQCM in finding gene co-expression modules.

### Data integration

As noted in [15], introducing prior-knowledge to network-based analysis may hold promise for comprehensively examining the interactions between genes underlying the pathogenesis of complex diseases. Fortunately, since there is a mass element, topology potential could integrate prior knowledge naturally. Compared with our proposed method, WGCNA and lmQCM cannot integrate prior knowledge when detecting co-expression modules. We weighted the nodes using the GDA scores provided by DisGeNET (https://www.disgenet.org/). The weights on the nodes resulted in less modules compared to not using prior knowledge. This result is due to the fact that the module identification process becomes more concentrated on genes that have stronger gene-disease associations. The TPSC identified two modules, G1 and G12, which had significant survival differences between patient groups. These two modules were both enriched in the cell cycle process. From the perspective of module merging, G1 merged part of M21 and M23 which are both cell cycle related survival significant modules detected by TPSC-1. In addition, module M1 was completed included in module G1. Although M1 was not survival significant, it also enriched to cell cycle processes. This fact revealed that by introducing prior knowledge, modules with similar functions trend to be merged. Module G12 mainly merged module M16 and part of M23 which have the strongest significance among all survival significant modules. Both M16 and M23 were survival significant modules detected by TPSC-1 but M16 did not significant enrich to any pathways related to cell cycle process. By introducing GDA information, we can infer that M16 is also a cell cycle related module. These results reflects the nature of prior knowledge, in which cell cycle-related genes have higher GDA scores and is known to be an essential biological process in cancer cells [27]. The results suggest that our proposed topology potential based method can filter out modules with deep biological meanings by introducing prior knowledge.

### Enriched cytobands

In cancer data, gene co-expression are highly confounded by locations due to extensive existence of CNVs. And we can take advantage of this phenomenon to infer potential CNV events from transcriptomic data. In this paper, since we began with CNV information, it is not surprising that some genes are located on the same cytoband. In Table 2, we can find some cytobands enriched in modules detected by different methods. Around these positions, some of them are frequently identified, such as chromosome 1p31-32, 1q31-32, 17q11[28–32].

## Conclusion

In this paper, we provided a novel approach for detecting co-expressed modules in gene co-expression network based on topology potential and spectral clustering algorithms. The method improved upon a previous method for full-connected network and asymmetric Laplacian matrix. By testing our method on the TCGA-BRCA dataset, modules with a significant difference in overall survival time were detected by our method to have better coverage on input genes, more balance on module size and finer granularity. In addition, by coding prior knowledge as node weights, our proposed method can identify more related modules. This allows researchers to detect pathways or cytobands affected by specific clinical information.

Though we only applied our algorithms on mining gene co-expression network, our proposed method is designed for general use in mining any fully-connected weighted network with weights on both edges and nodes.

## Methods

### Module detect algorithm

In this part, we introduce our proposed module detection method based on topology potential and spectral clustering method (TPSC). There are two variants of TPSC, i.e., TPSC-1 and TPSC-w. The only difference between them is whether the nodes have weights. Since they have same workflow, we will introduce them together. The TPSC method, as an improvement of the method provided by [16], can handle full-connected weighted networks. The steps of TPSC algorithm is displayed in Algorithm 1.
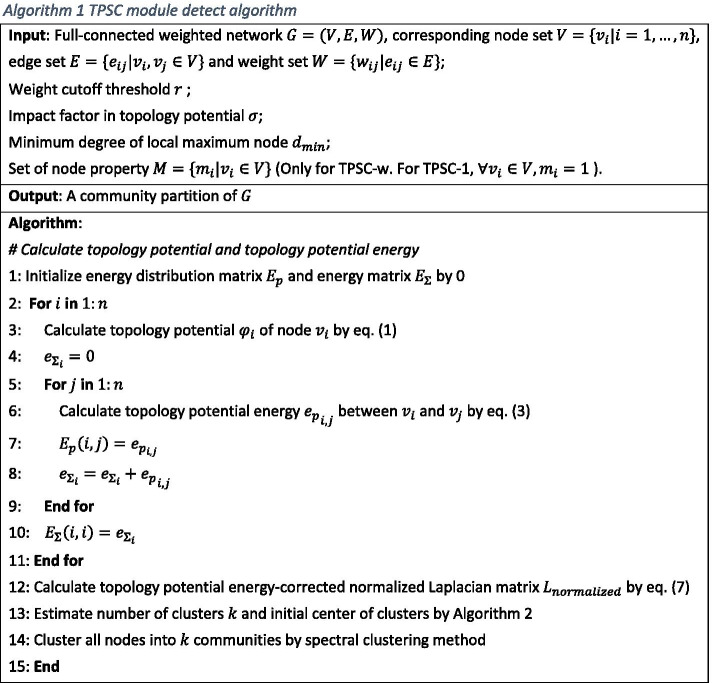


Compared with the original algorithm in [16], our method altered the technique of determining the number of clusters to make it suitable for fully connected weighted networks. In addition, we also adjusted the approach of normalizing Laplacian matrix to avoid the problem of asymmetry when node property is assigned. Next, we introduce the concept of topology potential.

#### Topology potential and energy

The theory of topology potential is a branch of field theory, which is used to describe the interaction and association among network nodes. Given a network $G\left( {V,E} \right)$, where $V = \{ v_{i} |i = 1, \ldots ,n\}$ is the set of nodes, $\left| V \right| = n$; $M = \{ m_{{v_{i} }} |v_{i} \in V\}$ is the set of mass of the nodes; and $E = \{ \left( {v_{i} ,v_{j} } \right)|v_{i} ,v_{j} \in V\}$ is the set of edges. Topology potential $\varphi$ of any node $v_{i} \in V$ can be defined as:

1 $$\begin{array}{*{20}c} {\varphi \left( {v_{i} } \right) = \mathop \sum \limits_{{v_{j} \in V\backslash \left\{ {v_{i} } \right\}}} m_{{v_{i} }} \times \exp \left( { - \left( {\frac{{d_{ij} }}{\sigma }} \right)^{2} } \right)} \\ \end{array}$$

where $d_{ij}$ is the distance between node $v_{i}$ and $v_{j}$. For fully-connected weighted networks, $d_{ij}$ can be calculated by weighting using appropriate methods such as inverse of correlation coefficient. $\sigma$ is an impact factor to control the influence range of nodes. Contribution of $v_{j}$ to topology potential of $v_{i}$ will decay to 0 rapidly if the distance between them is greater than $\frac{3\sigma }{{\sqrt 2 }}$ [33].

In the original method, the author proposed a concept called potential component to quantitatively describe the relationship between two nodes. Potential component produced by node $v_{i}$ at position $v_{j}$ is defined as:

2 $$\begin{array}{*{20}c} {p_{i,j} = m_{{v_{i} }} \times \exp \left( { - \left( {\frac{{d_{ij} }}{\sigma }} \right)^{2} } \right)} \\ \end{array}$$

Obviously, if $m_{{v_{i} }} \ne m_{{v_{j} }}$, $p_{i,j}$ will not equal to $p_{j,i}$. This violates the definition of a metric and will cause asymmetry of the normalized Laplacian matrix if the mass of each node is different. To solve this problem, we defined a novel metric, topology potential energy, to provide the same function as the potential component. Topology potential energy of node $v_{j}$ in the potential field of node $v_{i}$ is defined as:

3 $${e_{{p,i,j}} = m_{{v_{i} }} m_{{v_{j} }} \times \exp \left( { - \left( {\frac{{d_{{ij}} }}{\sigma }} \right)^{2} } \right)}$$

In particular, $e_{pi,j} = 0$ if $i = j$. If the mass of each node is not assigned (i.e. mass of all nodes equal to 1), $e_{pi,j}$ is equivalent to $p_{i,j}$.

#### Topology potential energy-corrected normalized Laplacian matrix

The Laplacian matrix can be treated as matrix representation of a network, which can be used to find many useful properties of that network. Traditionally, the Laplacian Matrix $L$ of simple network is defined as:

4 $$\begin{array}{*{20}c} {L = D - A} \\ \end{array}$$

where $D$ is degree matrix and $A$ is adjacency matrix. For weighted networks, $D$ is replaced by weighted degree matrix and $A$ is replaced by weight adjacency matrix. Its normalization version can be defined as:

5 $$\begin{array}{*{20}c} {L_{normalized} = D^{{ - \frac{1}{2}}} LD^{{ - \frac{1}{2}}} = I - D^{{ - \frac{1}{2}}} AD^{{ - \frac{1}{2}}} } \\ \end{array}$$

To add additional structural information to the network, we will redefine Laplacian matrix $L$ by replacing weighted degree matrix $D$ with weight adjacency matrix $A$. Compared with traditional Laplacian matrix, the redefined one transformed the weight of edges by topology potential and include the prior knowledge.

Weight adjacency matrix $A$ will be replaced by potential energy distribution matrix $E_{p}$. $E_{p}$ is an *n*-dimensional symmetry matrix. Diagonal element of $E_{p}$ is all zero and the other elements are potential energy of two nodes calculated by Eq. (3).

Weighted degree matrix $D$ is replaced by the total potential energy matrix $E_{{\Sigma }}$. $E_{{\Sigma }}$ is an *n*-dimensional diagonal matrix, whose diagonal element $e_{{{\Sigma }_{i} }}$ is the sum of topology potential energy of node $v_{i}$ in potential field of any other nodes. $e_{{{\Sigma }_{i} }}$ can be calculated by:

6 $$\begin{array}{*{20}c} {e_{{{\Sigma }_{i} }} = \mathop \sum \limits_{{v_{j} \in V}} e_{pi,j}= m_{{v_{i} }} \mathop \sum \limits_{{v_{j} \in V\backslash \left\{ {v_{i} } \right\}}} m_{{v_{j} }} \times \exp \left( { - \left( {\frac{{d_{ij} }}{\sigma }} \right)^{2} } \right)} \\ \end{array}$$

After all, topology potential energy-corrected normalized Laplacian matrix can be defined by:

7 $$\begin{array}{*{20}c} {L_{normalized} = I - E_{{\Sigma }}^{{ - \frac{1}{2}}} E_{p} E_{{\Sigma }}^{{ - \frac{1}{2}}} } \\ \end{array}$$

#### Determination of number of clusters

Number of clusters $k$ in spectral clustering can be determined by $k$ nontrivial eigenvalues whose eigenvector elements present a ladder distribution. However, if the community structure is not clear, the distribution of eigenvector elements will not show obvious ladders, which prevent us to get proper number of clusters[34]. In order to solve this problem, [16] provided a local maximum topology potential based method, but directly using this metric in a fully connected network is not possible since there is no local maximum topology potential nodes but a global one. We need downgrade the network to a simple one to search for local maximum nodes. This work can be done by hard-thresholding on the weight.

For co-expression networks, co-expressed nodes are all adjacent. Hence, we only focus on first order neighbors of nodes and not the higher order ones, which can be used to simplify the local maximum node search process. We display the pseudocode of this procedure in Algorithm 2. In our algorithm, we used a greedy-based method which selects for the maximum potential node and remove its neighbor iteratively. In addition, the degree of nodes can also be considered since nodes with low degrees contribute less to co-expression modules. The iteration process can be terminated early by degree threshold (step 14). Local maximum nodes can be treated as the initial center of a spectral cluster. Algorithm 2 displayed the pseudocode of determination of number of clusters.
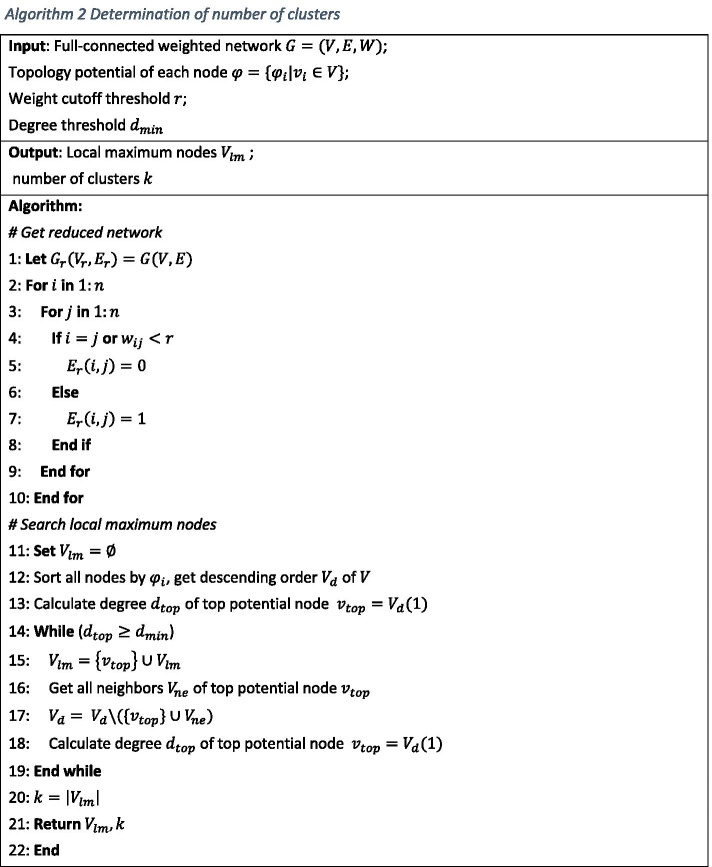


### Application of TPSC to gene expression data

BRCA gene expression data was obtained from TCGA. The dataset contained 1,098 cases with multiple types of samples, such as primary tumor, recurrent tumor and normal tissue etc. We selected 999 primary tumor samples with corresponding patient survival data for our analysis. For our pre-processing step, we removed genes with an expression value of 0 in more than 50% of the samples. Genes with the lowest 20% of mean values and lowest 10% variance were also be removed.

To identify disease related pathways, we selected genes that have an association with disease to construct gene co-expression network. These genes were obtained from the DisGeNET database [35], the largest publicly available collection of genes and variants associated with human diseases. In the DisGeNET database, Gene-disease associations (GDA) were ranked by a GDA score ranged from 0 to 1 that considers the number and type of sources (e.g., level of curation, organisms), as well as the number of publications supporting the association. For a pair of specific disease and gene, if GDA score between them is greater than 0, there exists a known gene-disease association. In DisGeNET, there are 4,962 genes reported to be associated with Breast Carcinoma (C0678222) and 4,125 of them were found in our gene expression data. We used genes with a GDA score greater than 0 in our analysis, and GDA score is used as weight of genes while using TPSC-w algorithm.

### Analysis of weighted gene co-expression network

We used Pearson correlation coefficients (PCCs) to construct a co-expression network. Specifically, we computed PCCs between each pair of genes as weight. The distance between two genes was defined by the inverse of their PCC. For TPSC-w, weights of genes were set as gene disease association scores provided by DisGeNET. Weight cutoff threshold was set to 0.3 as while larger PCC value indicates higher correlations between different genes. The threshold for degree was set to 5. We also performed comparative analysis with the well-known WGCNA and our previous lmQCM algorithm. All parameters in these two methods were set as recommended in the corresponding papers. Overlaps of modules detected by different algorithms were tested by Fisher’s exact test with the R package ‘GeneOverlap’ [36].

To assess the association between co-expression modules and patient survival information, overall survival (OS) analysis was performed based on the eigengene of modules. Eigengene was calculated to represent the expression of co-expressed gene modules, with a positive value defined as the module being up-regulated and a negative value defined as the module being down-regulated [37]. Thus, we can divide all the patients into two groups by the sign of eigengene entries for each module. Then the Kaplan–Meier estimator was used for patient stratification and log-rank test was applied to compare the survival difference between two groups with the R package ‘survminer’ [38].

The biological relevance of the network modules was obtained by carrying out enrichment analysis using the online tool ToppGene [39]. We focused on the modules that showed significant survival difference between patient groups and used the ToppGene suites to find significantly enriched gene oncology (GO) terms, pathways, and cytobands that are associated with these survival significant modules.

## Supplementary Information

**Additional file 1:** Kaplan-Meier curve of module (a)M6, (b)M16, (c)G1, (d)G12,(e)W13 and (f)Q7.

**Additional file 2:** Detailed result of biological process, pathway and cytoband enrichment on each module. Only items with top 5 enrichment significance and number of overlap genes are displayed in this table.

## Data Availability

The TCGA data used in this study is available in Broad GDAC Firehose (https://gdac.broadinstitute.org/). Gene disease association data used in this study is available in DisGeNet (https://www.disgenet.org/).

## Abbreviations

TPSC: topological potential based spectral clustering
PCC: pearson correlation coefficient
PPI: protein–protein interaction
BRCA: breast cancer
TCGA: the Cancer Genome Atlas
OS: overall Survival
B&H FDR: benjamini and Hochberg false discovery rate
