# Supplementary figures and images for: TPSC: a module detection method based on topology potential and spectral clustering in weighted networks and its application in gene co-expression module discovery
Source: BMC Bioinformatics. 2021 Oct 25;22(Suppl 4):111. doi: 10.1186/s12859-021-03964-5 (PMC8543836; doi:10.1186/s12859-021-03964-5)

(a)M6

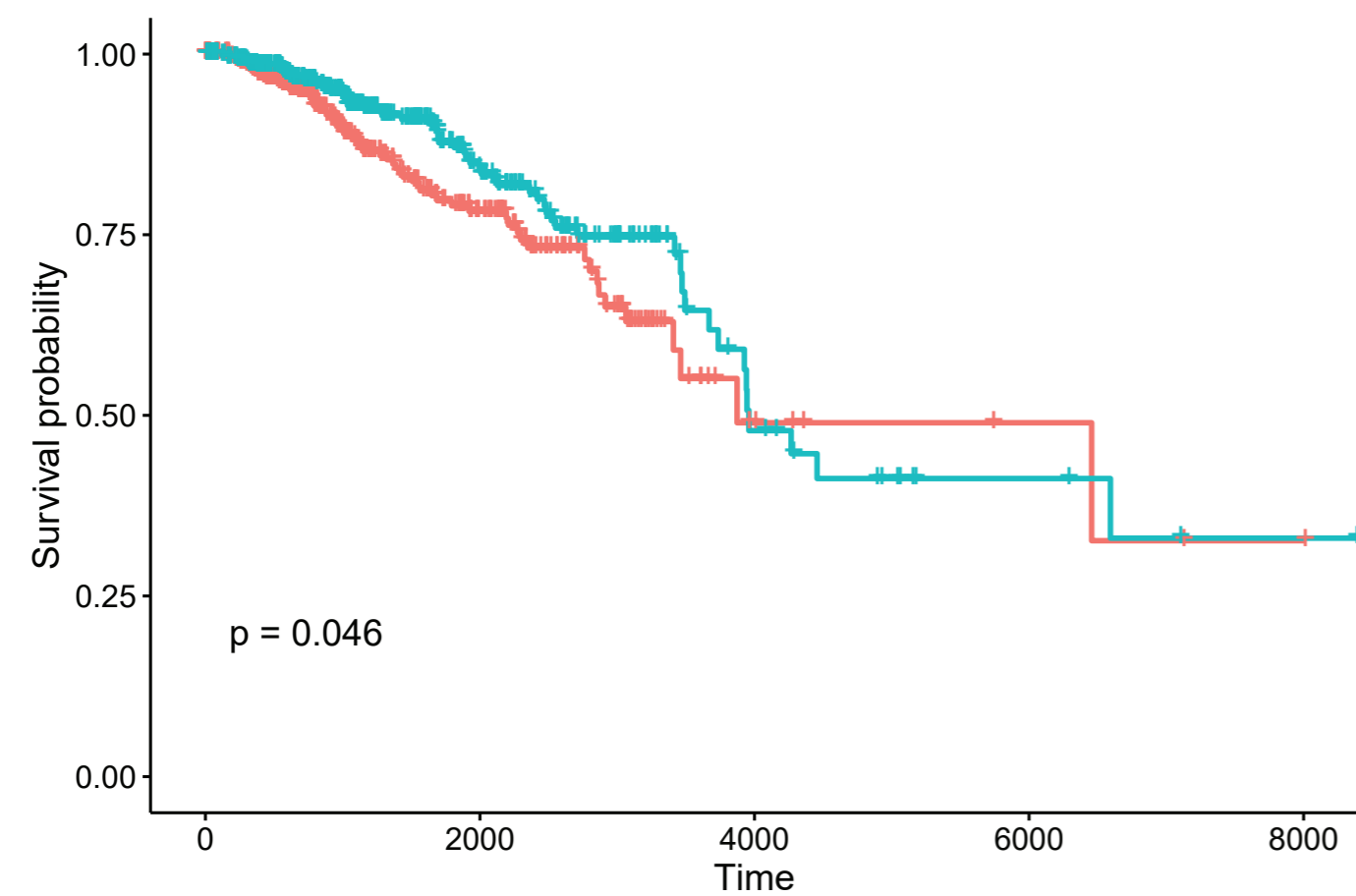

(b)M16

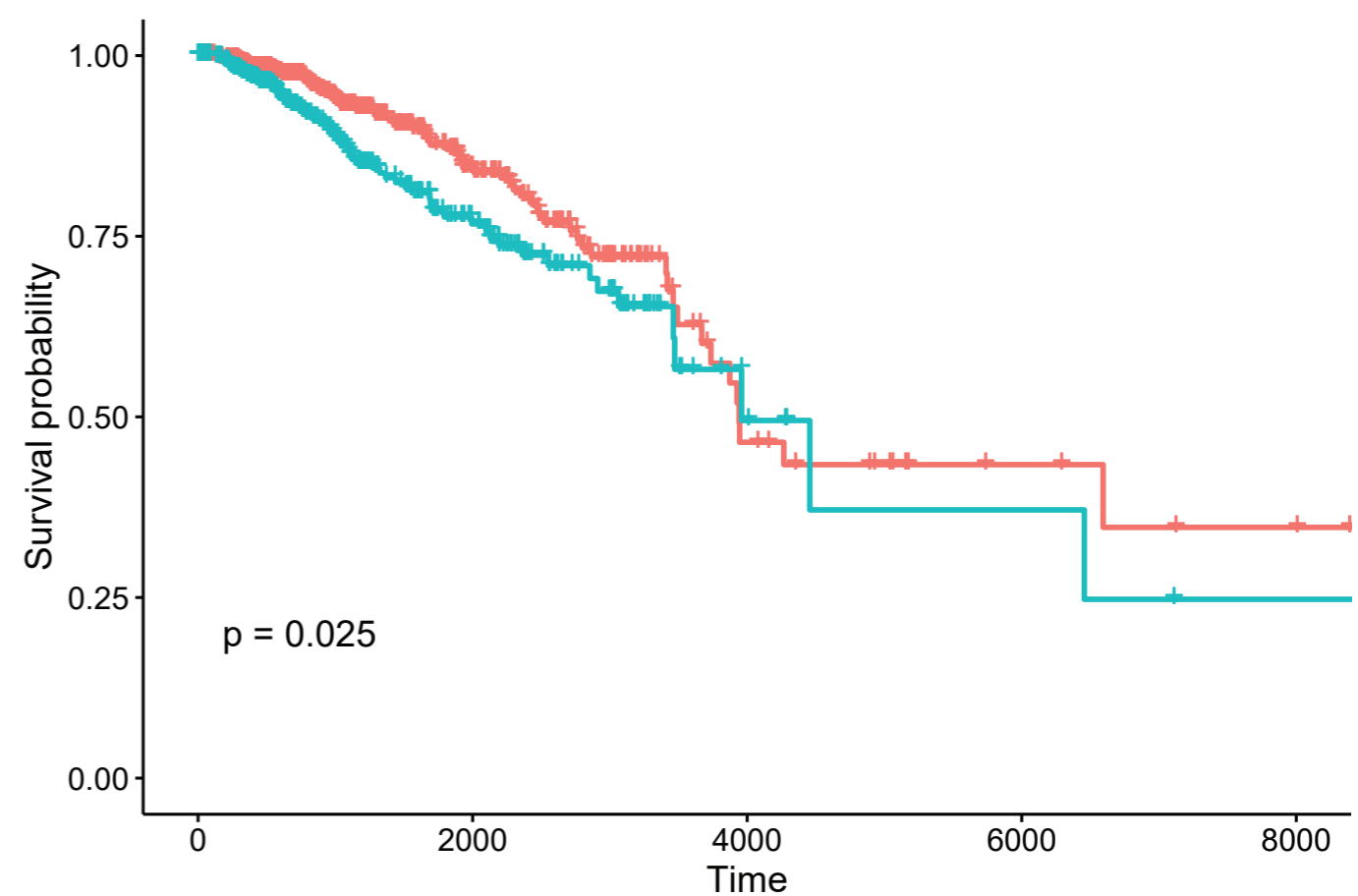

(c)G1

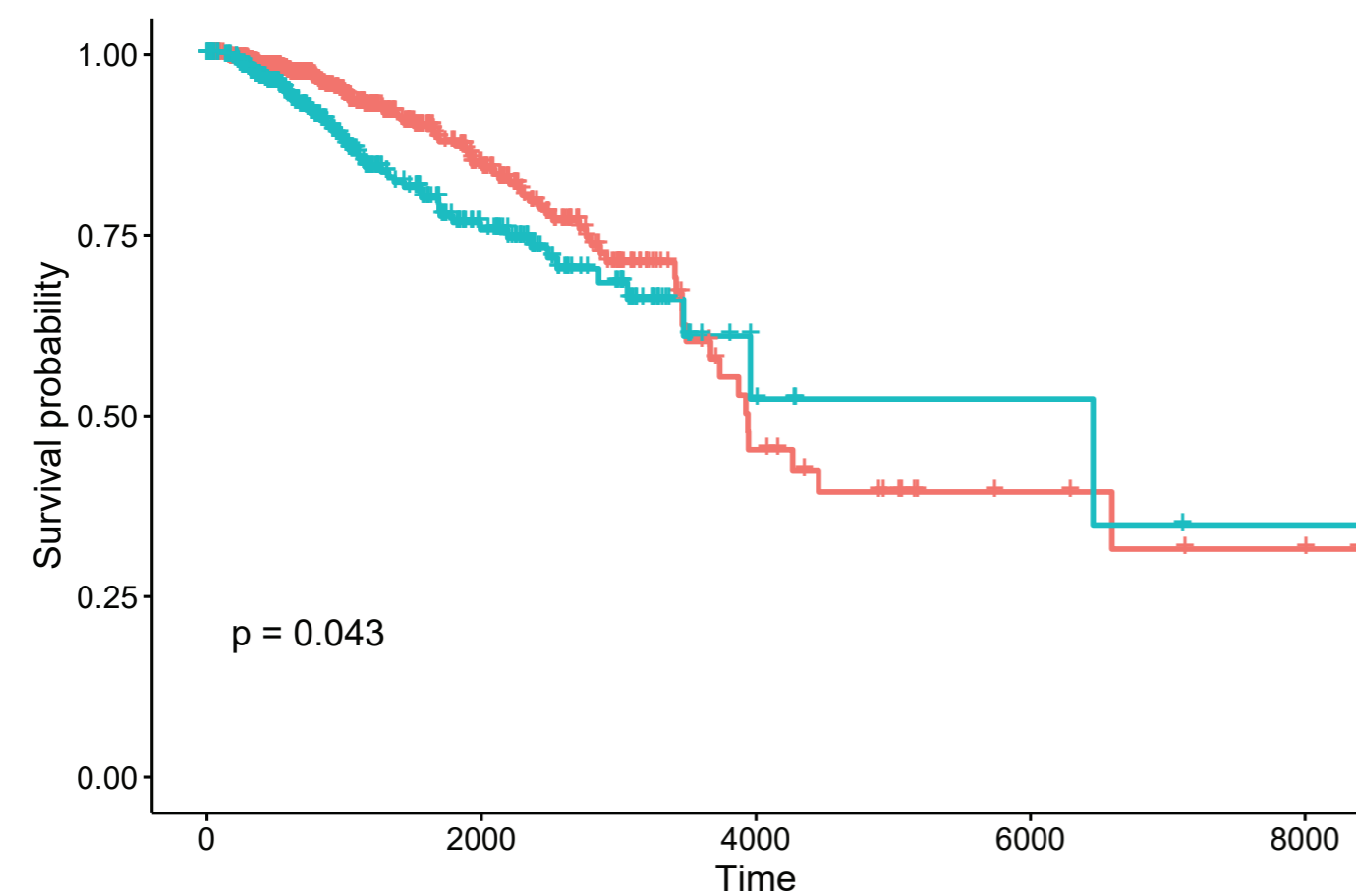

(d)G12

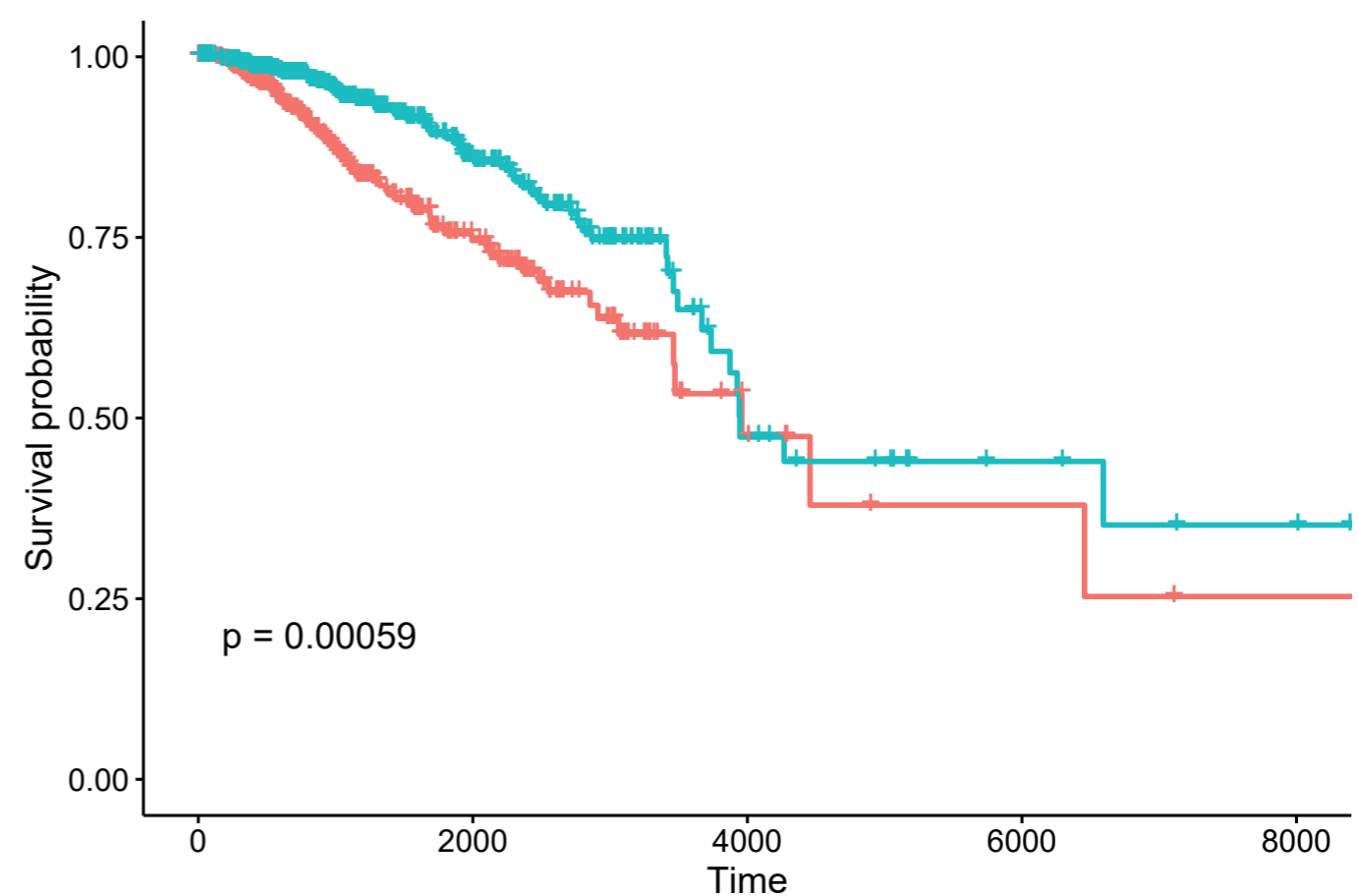

(e)W13

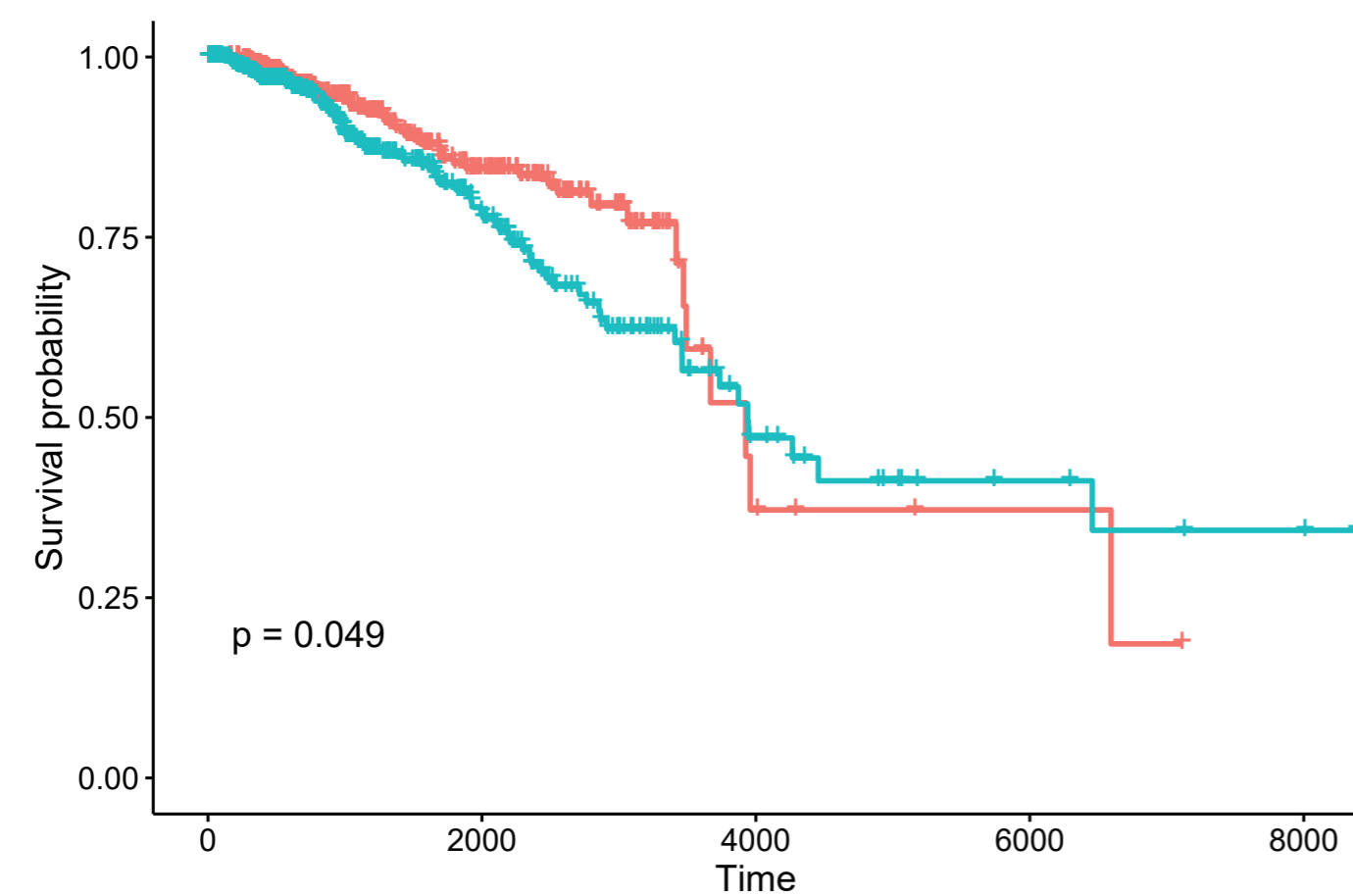

(f)Q7

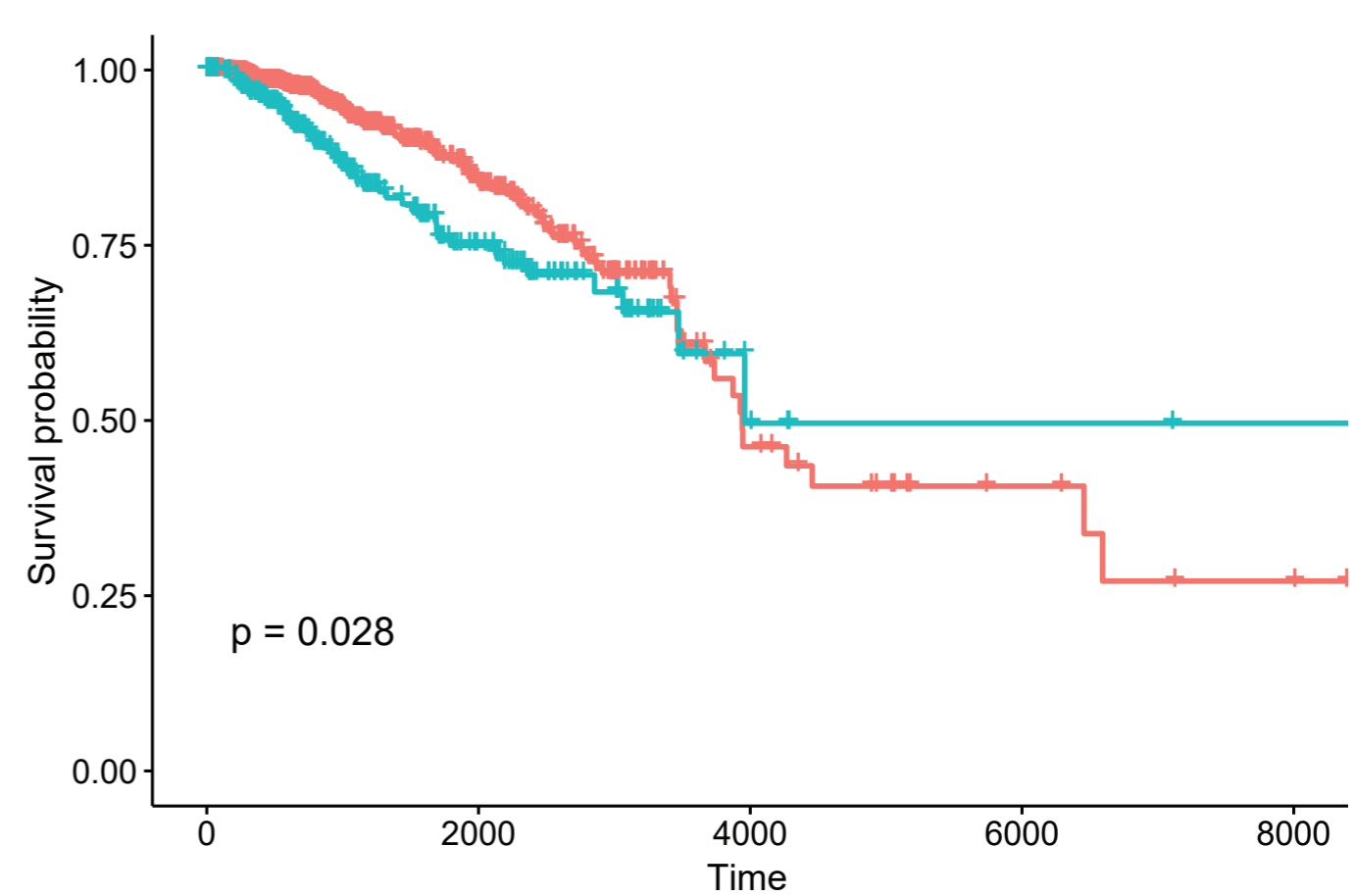

+ Down-regulated

+ Up-regulated

Supplement: Supplementary file 1 — Additional file 1: Kaplan-Meier curve of module (a)M6, (b)M16, (c)G1, (d)G12,(e)W13 and (f)Q7. [file 12859_2021_3964_MOESM1_ESM.pdf]
